# Supplementary figures and images for: The circadian clock is disrupted in pancreatic cancer
Source: PLoS Genet. 2023 Jun 1;19(6):e1010770. doi: 10.1371/journal.pgen.1010770 (PMC10263320; doi:10.1371/journal.pgen.1010770)

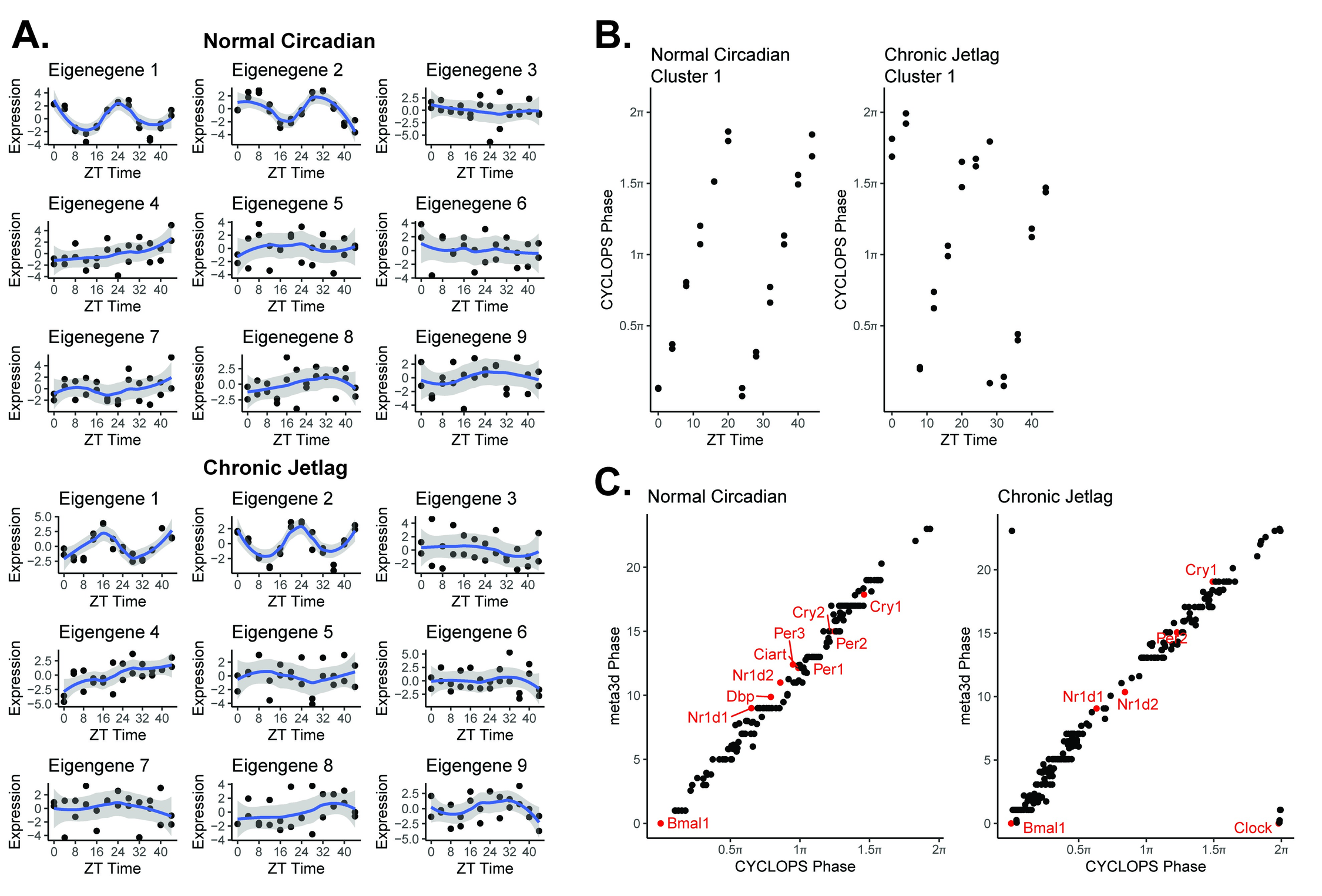

Supplement: S1 Fig — Eigengenes identified by Oscope for use in CYCLOPS in A. normal circadian (upper) and chronic jetlag (lower) samples. Shading around the blue regression line indicates the 95% confidence interval for each plot. B. The normal circadian (left) and chronic jetlag (right) clusters ordered by CYCLOPS demonstrate accurate reordering for both conditions C. Normal circadian (left) and chronic jetlag (right) genes found to be significantly rhythmic on both CYCLOPS reordered cosinor analysis and rhythmicity testing based on the known sample collection time with the Metacycle meta3d function are ordered by their predicted phase of expression. Clock genes are shown in orange. (TIF) [file pgen.1010770.s001.tif]

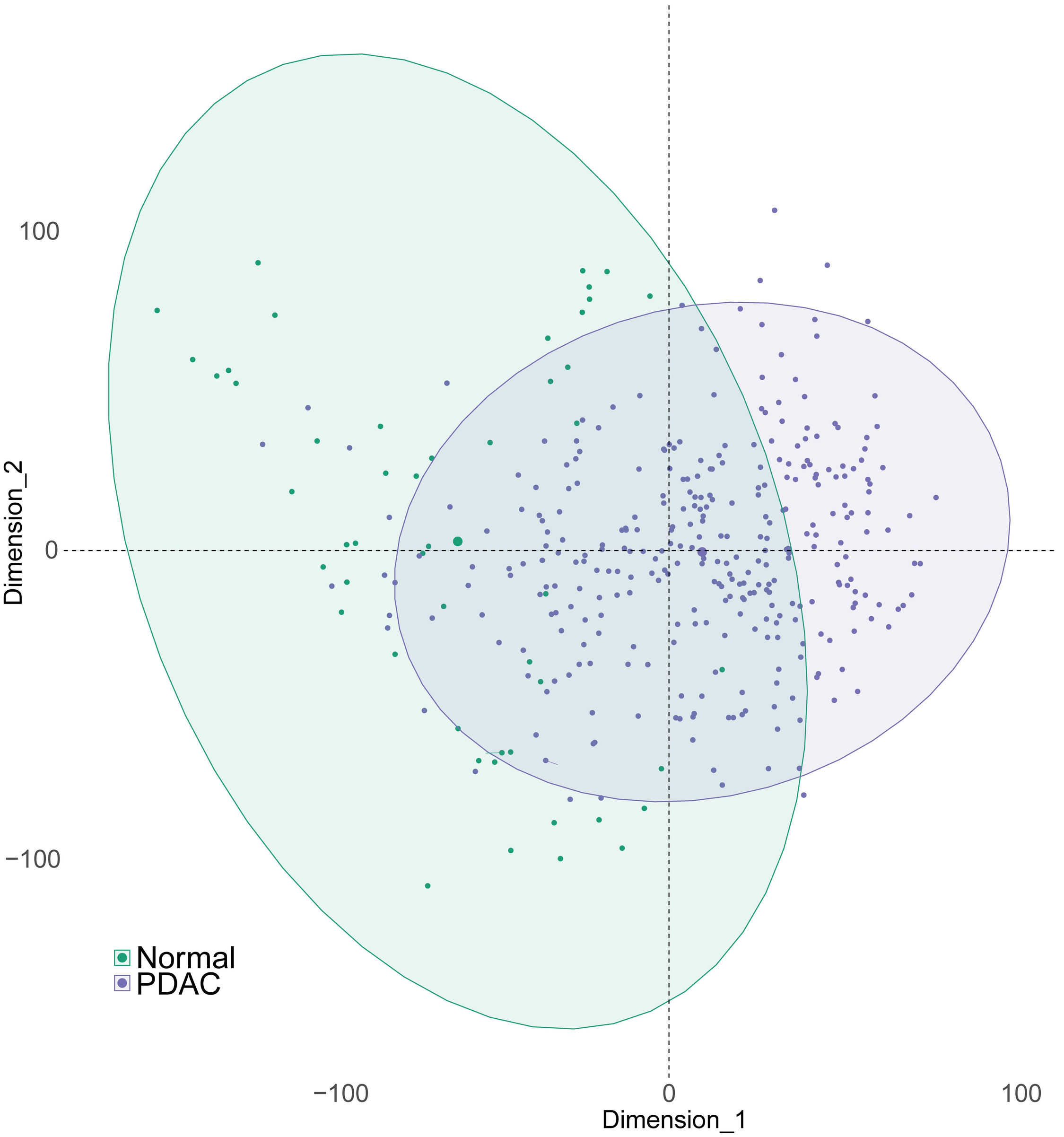

Supplement: S2 Fig — TCGA and CPTAC-3 samples were processed and batched corrected. An MDS plot is shown demonstrating differences between the matched normal (green; n = 50) and PDAC (purple; n = 318) samples. (TIF) [file pgen.1010770.s002.tif]

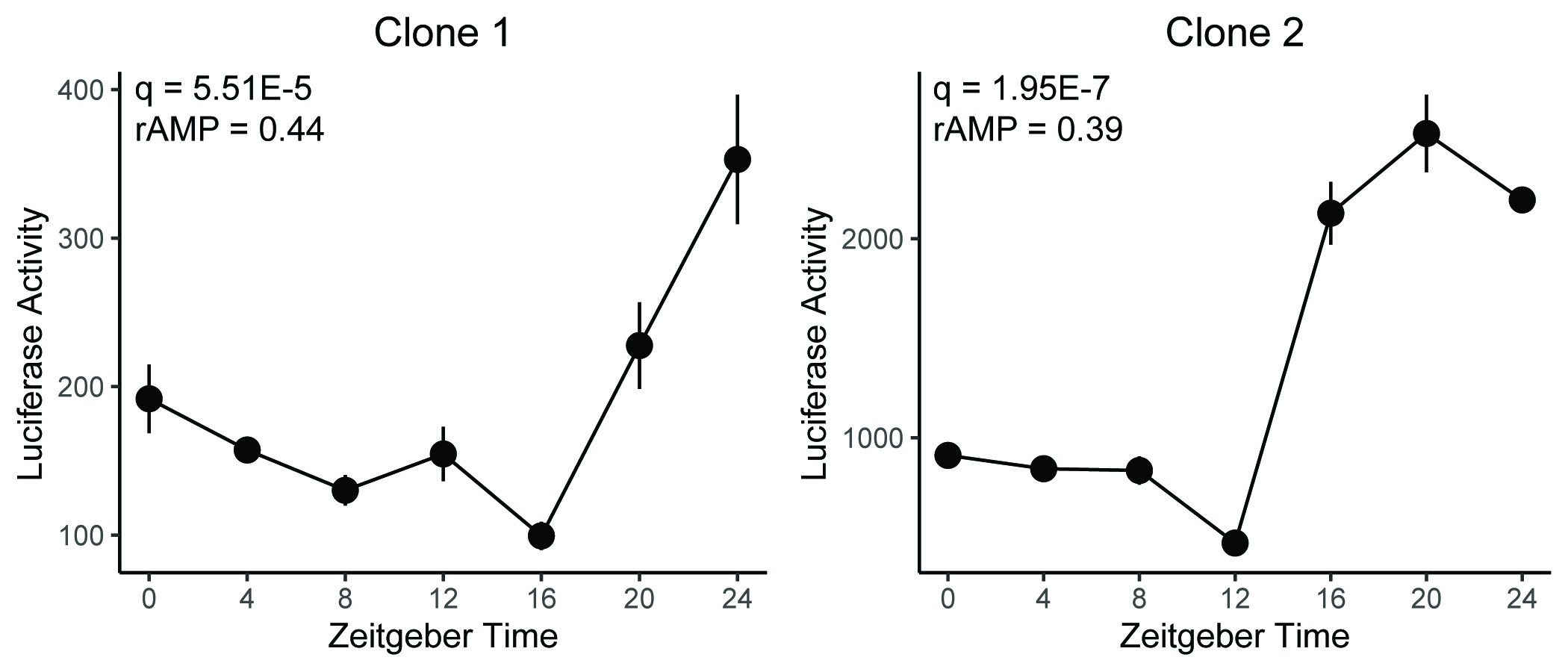

Supplement: S3 Fig — Two independent clones were examined, and luciferase activity was measured at 4-hour intervals (n = 6 per time point). Rhythmicity was calculated with Metacycle. Clone 1 (left) was found to be rhythmic with a q = 5.51E-5 and rAMP = 0.44. Clone 2 (right) was found to be rhythmic with a q = 1.95E-7, rAMP = 0.39. (TIF) [file pgen.1010770.s003.tif]

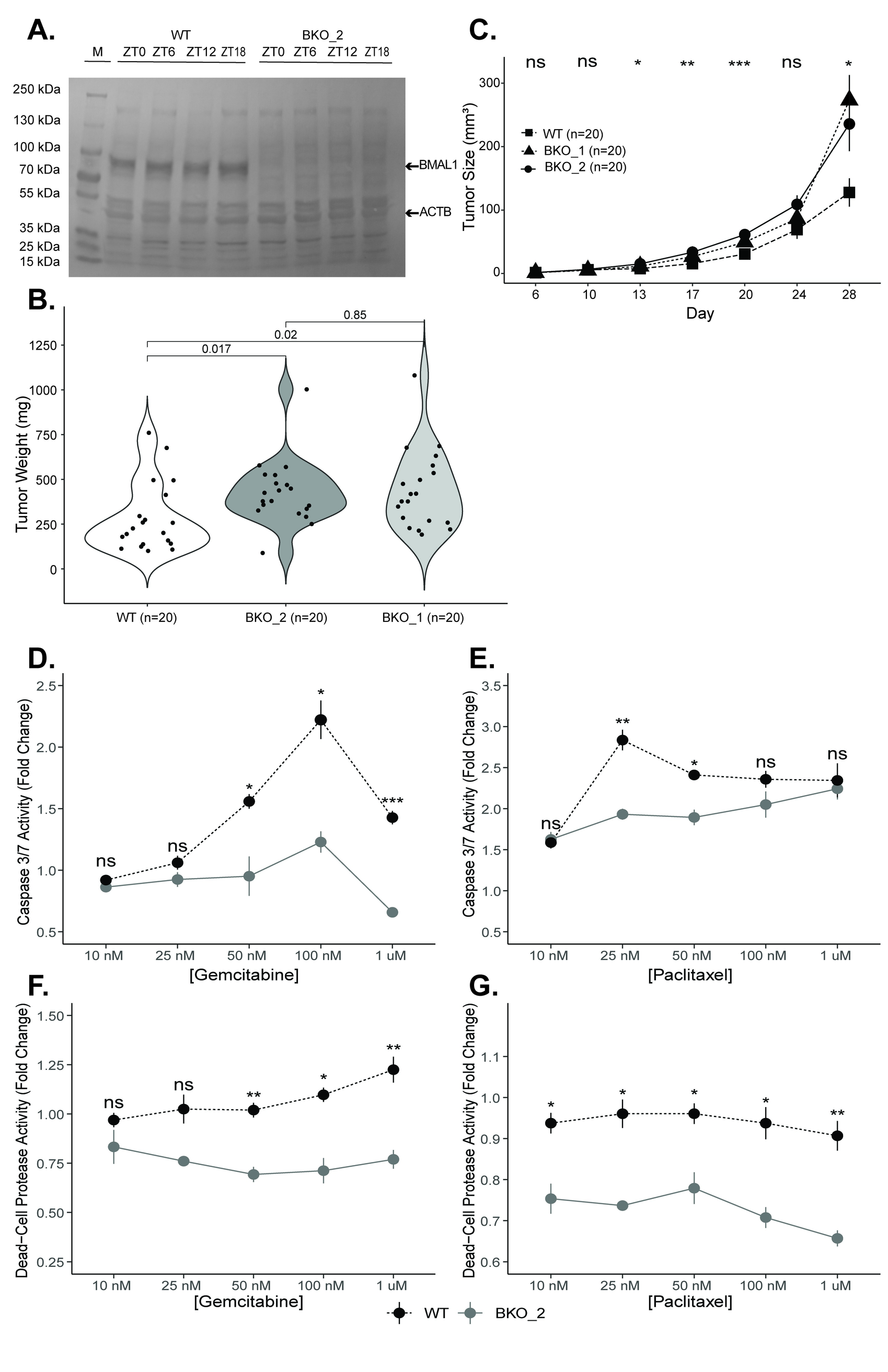

Supplement: S4 Fig — A second independent Bmal1 knockout (BKO) clone with an identical mutation to the first was validated with A. western blot analysis. We then heterotopically implanted wildtype (WT) and BKO cells into C57BL/6J mice and followed B. tumor weight and C. cell growth. BKO_2 (dark grey; n = 20) had a similar mean (± standard error) tumor weight to BKO_1 (light grey; n = 20) (426.09 (± 40.07) mg vs 438.02 (± 48.84) mg; p = 0.85), and both BKO_2 (426.09 (± 40.07) mg vs 280.11 (± 42.73) mg; p = 0.017) and BKO_1 (438.02 (± 48.84) mg vs 280.11 (± 42.73) mg; p = 0.02) were larger than WT (white; n = 20). BKO_1 and BKO_2 had similar significantly faster growth trajectories. D-G. KPC wildtype (WT) and BKO_2 cells (n = 3) were treated with increasing doses of gemcitabine and paclitaxel. Fold change differences (± standard error) in Caspase 3/7 activity in response to either D. gemcitabine or E. paclitaxel. Fold change differences (± standard error) in dead-cell protease activity in response to F. gemcitabine or G. paclitaxel. Comparisons between conditions at each concentration were made with t-test. [ns = not significant, * = p < 0.05, ** = p < 0.01, *** = p < 0.001]. (TIF) [file pgen.1010770.s004.tif]
